# Is coronal imbalance in degenerative lumbar scoliosis patients associated with the number of degenerated discs? A retrospective imaging cross-sectional study

**DOI:** 10.1186/s12891-023-06558-9

**Published:** 2023-05-25

**Authors:** Nanshan Ma, Xiangsheng Tang, Wenhao Li, Zhencheng Xiong, Wenhai Yan, Jiaojiao Wang, Tianwen Gu, Mingsheng Tan

**Affiliations:** 1 Beijing University of Chinese Medicine, Beijing, 100029 People’s Republic of China; 2 Department of Orthopaedic Surgery, China-Japan Friendship Hospital, 100029 Beijing, People’s Republic of China; 3 Department of Orthopaedic Surgery, Dongzhimen Hospital, 100029 Beijing, People’s Republic of China; 4 Department of Orthopaedic Surgery, West China Hospital, 610041 Chengdu, People’s Republic of China

**Keywords:** Degenerative lumbar scoliosis, Coronal imbalance, Degenerated disc, Retrospective study

## Abstract

**Background:**

Degenerative lumbar scoliosis (DLS) is a common degenerative disease of the spine, that predominates in the elderly, and causes spinal deformities along with severe pain and reduced quality of life. The relationship between DLS and degenerated discs is now a new direction of research. Our study aimed to the relationship between the imaging parameters of coronal imbalance and the number of degenerated discs in patients with degenerative lumbar scoliosis and analyzed the segmental distribution of the degenerated discs in patients with DLS.

**Methods:**

We performed a retrospective analysis of the imaging of 40 patients who met the inclusion criteria who attended our outpatient clinic between April 2021 and July 2021, measuring the intervertebral space height of the AV (high side and low side), Cobb angle, and AVT (Apical vertebral translation) from coronal X-ray. Degenerated discs were evaluated by the Pfirrmann score based on T2-weighted magnetic resonance images. We record the number of degenerated discs (Graded as Grade III, Grade IV or Grade V by the Pfirrmann score) and the segments in which they are located. Finally, we explore the relationship between the imaging parameters of coronal imbalance and the number of degenerated discs in patients with DLS.

**Result:**

Among the 40 patients with DLS in our study, all patients had degenerated discs in the lumbar spine, 95% of patients had degenerated discs(Pfirrmann score Grade III, Grade IV or Grade V) in 2 or more segments, with the L4-L5 segment being the most involved segment with the most degenerated discs, followed by the L3-L4 segment and the L5-S1 segment. There was no statistically significant relationship between the number of degenerated discs and the coronal imbalance in patients with DLS.

**Conclusion:**

Our results showed an association between DLS and degenerated discs, but there was no statistically significant relationship between imbalance in the coronal plane of the lumbar spine and the number of degenerated discs in patients with DLS. The distribution of degenerated disc segments in patients with DLS showed a higher likelihood of disc degeneration in 2 or more segments, and a higher frequency of disc degeneration in the inferior disc and in the adjacent segments of the AV.

## Introduction

Degenerative lumbar scoliosis (DLS) is a common degenerative disease of the spine, which differs from congenital scoliosis in that it is often caused by degeneration of the lumbar discs and small joints, resulting in lumbar instability and scoliosis, and the prevalence of DLS in the elderly is approximately 8.7% [1, 2]. The patient’s low back pain, radiating pain in the lower extremities and intermittent claudication are difficult to relieve if the lumbar imbalance is not improved, the deformity corrected, and the normal spinal canal structure restored through surgical treatment. The degree of scoliosis may increase over the years, eventually causing unavoidable harm [3].

It is well known that degeneration of lumbar discs is an important cause of coronal deformities in patients with DLS [4–6]. In clinical practice, patients with DLS often encounter degenerated discs in multiple segments, therefore, the relationship between coronal imbalance and the number of degenerated discs in patients with DLS is of great importance. We designed this study to discuss the relationship between the imaging parameters of coronal imbalance and the number of degenerated discs in patients with degenerative lumbar scoliosis and analyzed the segmental distribution of the degenerated discs.

## Materials and methods

### Subject

This study retrospectively analyzed 40 patients with DLS (29 cases female, 11 cases male), aged 40–83 years (mean age: 52.1 ± 11.9 years) who attended our outpatient clinic between April 2021 and June 2021(Characteristic of patients in this study is shown in Table 1). Inclusion criteria were:1) Included patients were > 40 years of age and had a coronal Cobb angle > 10°; 2) Included patients completed lumbar spine X-ray and lumbar spine T2-weighted magnetic resonance imaging (MRI) in a standing position. Exclusion criteria included (1) Patients with a previous diagnosis of idiopathic or congenital spinal deformity, spinal tumor, spinal tuberculosis, lumbar compression fracture, connective tissue disease, rheumatoid arthritis, and ankylosing spondylitis; (2) Patients with any previous history of lumbar spine surgery; (3) Patients with a history of lumbar trauma within 3 months prior to admission. The study was approved by all patients included in the study.

**Table 1:** Characteristic of patients in this study

Sex (M: F)	Age	Height (cm)	Weight (kg)	Course of disease(year)
11:29	52.1 ± 11.9	162.5 ± 8.2	68.4 ± 15.8	9.4 ± 8.2

### Imaging measurement methods

All patients were asked to complete the lumbar spine x-ray in a standing position. And to ensure the accuracy of the patient’s imaging results, pain medication is given to patients who are unable to maintain a standard posture for imaging due to factors such as root irritation to prevent compensatory scoliosis deformities. After acquiring the patient’s lumbar spine radiographs and T2-weighted magnetic resonance images (MRI) of the lumbar spine, we measured the imaging data on the findings. Measurements of coronal balance parameters were performed independently by two doctors experienced in spinal surgery and their measurements were pooled and judged by a third doctor, with a third measurement if the difference was large (greater than 10%) and an average if the difference was small (less than 10%).

First, we determined three parameters: (1) End Vertebrae (EV), the vertebra with the greatest angle of inclination on the cephalad and caudal side of the lumbar lateral bend on the lumbar spine radiographs. (2) Center Sacral Vertical Line (CSVL), the line passing through the midpoint of the upper endplate of the S1, is perpendicular to the horizontal ground. (3) Apical Vertebrae (AV), the vertebra in scoliosis that is furthest from the CSVL, which can sometimes be an intervertebral disc.

**Fig. 1:**
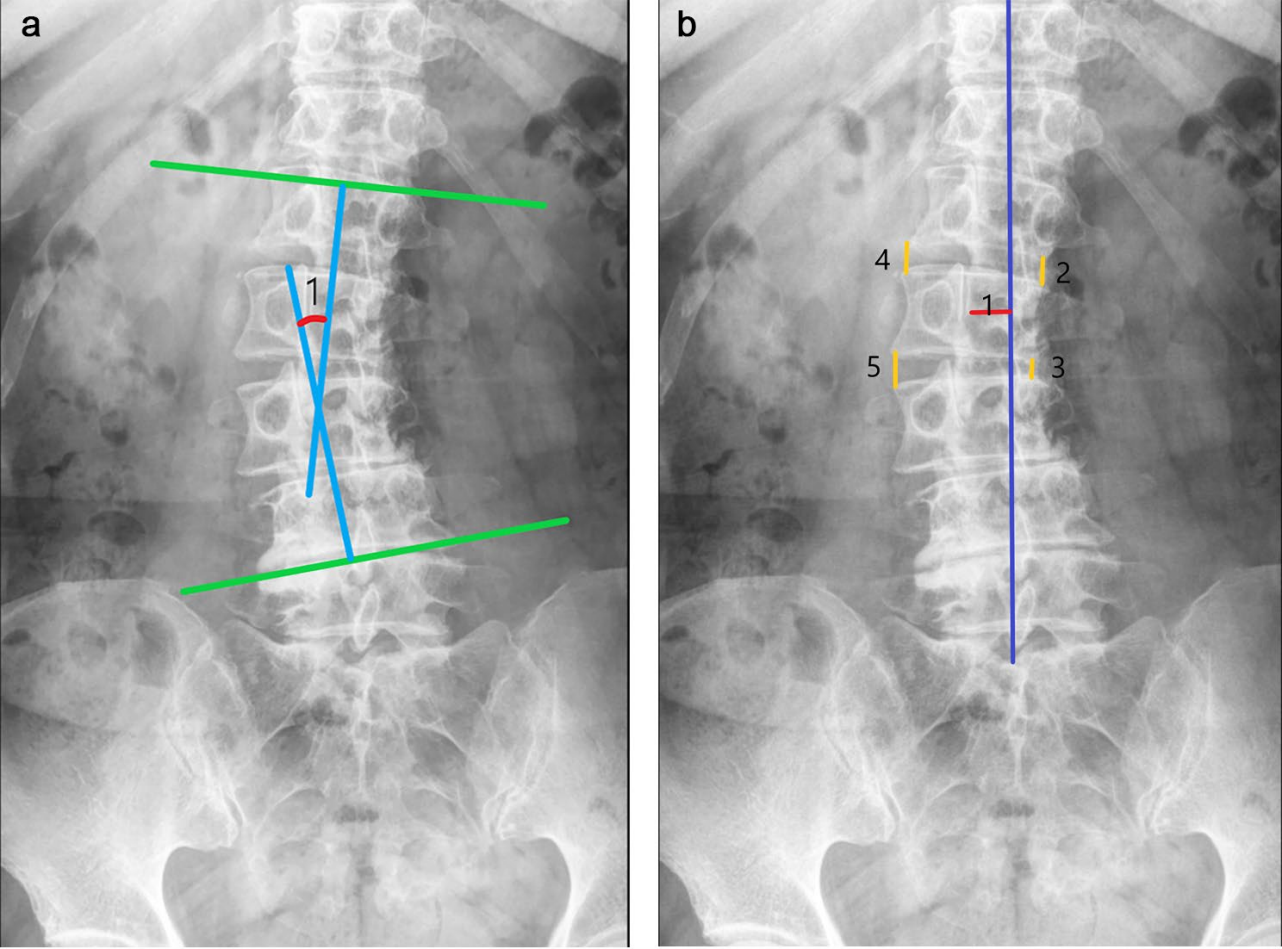
Imaging measurement methods

Subsequently, we measured four imaging parameters: Intervertebral space height of the AV (high side and low side), Cobb angle, and AVT (Apical vertebral translation) in 40 patients with DLS. The specific measurement methods are as follows (shown in Fig. 1).

*Intervertebral space height of the AV (high side and low side): The height of the intervertebral space in the upper and lower segments of the AV is measured on the coronal x-ray and averaged, divided into high side and low side. If the AV is an intervertebral disc, the high side height and low side height of the intervertebral space of that segment are taken.

* Cobb angle: When two lines are drawn on the coronal x-ray from the upper endplate of the superior EV and the lower endplate of the inferior EV, the angle between the two lines, or the angle between their perpendiculars, is known as the Cobb angle.

* AVT (Apical vertebral translation): the horizontal distance from the midpoint of the AV on the coronal x-ray to the CSVL.

In Fig. 1**(a)**, the patient’s EV is L1 and L4 and AV is L2, so angle 1 is the COBB angle for this patient. The blue line in Fig. 1**(b)** is CSVL, so line 1 is AVT and half the sum of line 2, line 3 and the sum of line 4, line 5 are the Intervertebral space height of the AV (low side and high side)

**Fig. 2:**
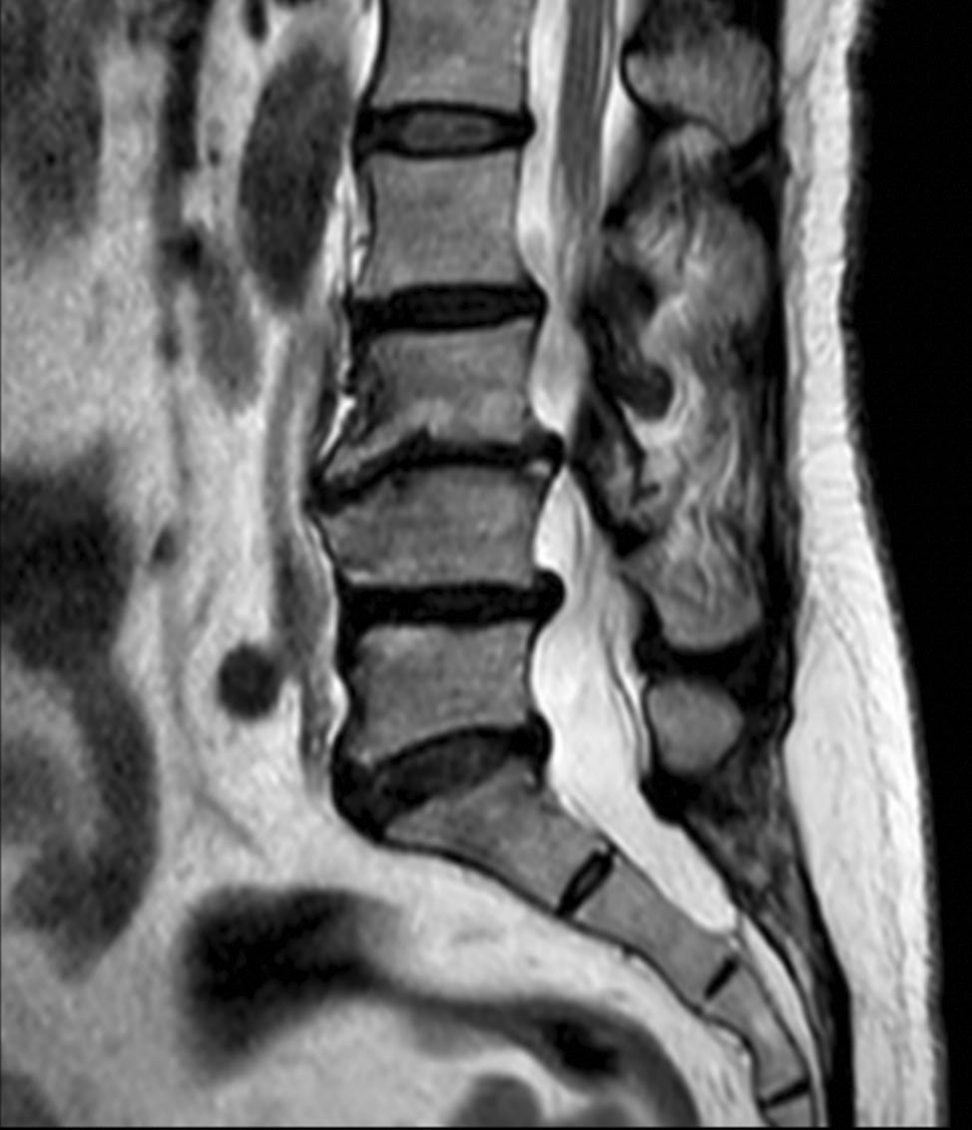
Degenerated discs evaluated by the Pfirrmann score.

Degenerated discs were evaluated by the Pfirrmann score based on T2-weighted magnetic resonance images. We record each patient’s number of degenerated discs (Grade III, Grade IV or Grade V by the Pfirrmann score), shown in Fig. 2. (Grade 3: uneven grey colour of the nucleus pulposus, no clear demarcation between the nucleus pulposus and the annulus fibrosus and slight reduction in disc height. Grade 4: uneven grey and black nucleus pulposus, disappearance of the boundary between the nucleus pulposus and the annulus fibrosus, moderate reduction in disc height. Grade 5: uneven black nucleus pulposus, loss of boundary between nucleus pulposus and annulus fibrosus, severe reduction in disc height.)

In Fig. 2, Grade III: L5-S1 intervertebral disc, Grade IV: L2-L3 intervertebral disc, L4-L5 intervertebral disc, Grade V: L3-L4 intervertebral disc.

### Data analysis

This study was statistically analyzed using SPSS 22.0 statistical software. The relationship between the number of degenerated discs and imaging parameters in patients with DLS was analyzed using Spearman Rank Correlation Analysis. Patient characteristics, as well as imaging parameters, were expressed as mean ± SD. In this study, P < 0.05 was considered statistically significant.

## Result

**Fig. 3:**
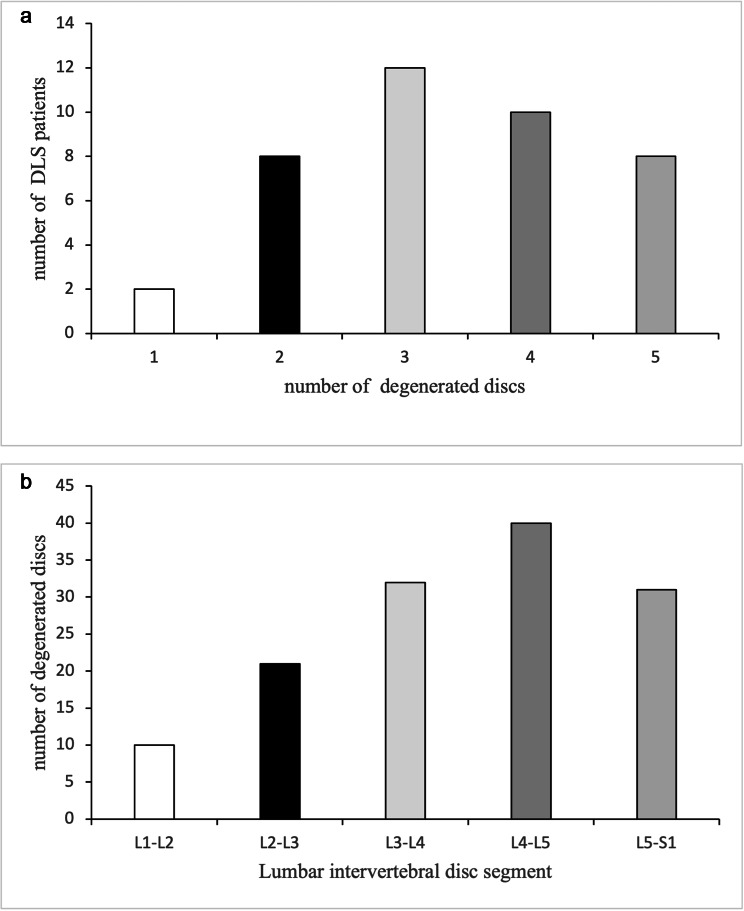
**(a)** The relationship between the number of degenerated discs and the number of patients. **(b)** The relationship between different intervertebral lumbar disc segments and the number of degenerated discs

Of the 40 patients with DLS included in this study, all patients had degenerated disc in the lumbar spine (degenerated disc graded as Grade III, Grade IV or Grade V by the Pfirrmann score). 2 patients (5%) had degenerated disc in a single segment, 8 patients (20%) had degenerated discs in 2 segments, 12 patients (30%) had degenerated discs in 3 segments, 10 patients(25%) had degenerated discs in 4 segments, and 8 patients(20%) had degenerated discs in all 5 segments. After counting the segments where all the degenerated discs were in the 40 patients with DLS, we found that a total of 134 segments of the lumbar discs were degenerated in the 40 patients. Of these, 10 segments (7.5%) were L1-L2, 21 segments (15.7%) were L2-L3, 32 segments (23.9%) were L3-L4, 40 segments (29.9%) were L4-L5, and 31 segments (23.1%) were L5-S1. The specific data are shown in Table 2 and the exact distribution is shown in Fig. 3a and 3b.

We performed Spearman Rank Correlation Analysis between the number of degenerated discs (degenerated disc graded as Grade III, Grade IV or Grade V by the Pfirrmann score) and lumbar spine radiographic parameters in 40 patients with DLS with SPSS 22.0. The intervertebral space height of the AV (high side and low side), Cobb Angle, and AVT (Apical vertebral translation) are 7.61 ± 2.6 mm, 4.9 ± 1.82 mm, 15.56 ± 6.06°, 7.83 ± 3.54 mm. The number of degenerated discs was not statistically related to the intervertebral space height of the AV (high side and low side), Cobb Angle, and AVT (p > 0.05) shown in Table 3.

**Table 2:** Number of degenerated discs after counting

Lumbar intervertebral disc segment	Number of degenerated discs(I)	Number of degenerated discs(II)		Number of patients
L1-L2	10	1		2
L2-L3	21	2		8
L3-L4	32	3		12
L4-L5	40	4		10
L5-S1	31	5		8

(I): Number of all degenerated discs in the different lumbar disc segments for all patients

(II): Number of degenerated discs per patient in this study

**Table 3:** Correlation Between the number of degenerated discs and lumbar spine radiographical coronal parameters

	Lumbar intervertebral space (high side, mm)	Lumbar intervertebral space (low side, mm)	COBB Angle	AVT (mm)
Mean	7.61	4.9	15.56	7.83
SD	2.6	1.82	6.06	3.54
Coefficient	0.048	-0.45	0.12	0.225
P value	0.771	0.782	0.461	0.162

## Discussion

The incidence of adult spinal deformity (ASD) is more than 60% of people over 60 years old and degenerative lumbar scoliosis is one of the most common types of ASD in adults. Several contemporary studies have shown that spinal imbalance in patients with DLS is closely related to a decline in mobility and quality of life [8–10]. In recent years, the incidence of DLS has been increasing and the symptoms it causes, such as neurogenic low back pain, leg pain, and intermittent claudication, have a serious impact on the quality of life of the middle-aged and elderly population [11].

DLS was earlier thought to be associated with metabolic bone diseases, such as osteoporosis. It has been suggested that scoliosis is caused by a collapse of the vertebral body due to post-stress changes in the vertebral body as a result of a microfracture of the trabeculae [12]. Robin et al. conducted a 7–13-year x-ray follow-up study of 554 subjects aged 50–84 years to investigate the appearance, presence, and progression of scoliosis in the elderly and its relationship with osteoporosis and back pain. The follow-up results showed no direct relationship between the presence or progression of scoliosis and osteoporosis [13]. The relationship between DLS and degenerated discs is now a new direction of research. In a 12-year prospective study of 60 volunteers, Kobayashi et al. found that the risk of degenerative lumbar scoliosis was greater when the difference in intervertebral space height was greater than 20% or when vertebral marginal osteophytes were greater than 5 mm. Kobayashi hypothesized that asymmetric intervertebral height changes cause small joints, vertebral marginal osteophytes, and distortion of the vertebral arch, followed by rotational subluxation or lateral slippage of the vertebral body, causing the lumbar spine to develop a lateral curvature in the coronal plane, and in the sagittal plane, it shows a loss of anterior lumbar protrusion and segmental retrognathism [4]. Yu et al. took intact discs intraoperatively from patients with lumbar scoliosis and performed immunohistochemical analysis, showing that normal discs have a large number of ordered elastic fibrous networks, whereas this elastic fibrous network is sparse and disrupted in scoliosis discs, suggesting that degeneration of the disc leading to disruption of the elastic fibrous network may be associated with the progression of spinal deformity [14]. In clinical practice, patients with DLS often encounter degenerated discs in multiple segments, therefore, we design this study to examine the relationship between the segmental distribution of degenerated discs and the number of degenerated discs and lumbar coronal imbalance in patients with DLS.

In our study, we selected the Intervertebral space height of the AV (high side and low side), COBB Angle, and AVT as indexes of the degree of DLS scoliosis. As mentioned above [4], asymmetric disc height degeneration is an important factor in inducing the formation of lumbar scoliosis, and AV is the vertebra with the greatest degree of deflection in DLS patient, so we selected the intervertebral space height of the AV as one of the indicators of DLS scoliosis progress, through which we measured the intervertebral space height of the AV (high side and low side) and analyze the relationship between it and the number of degenerated discs, we can indirectly observed the relationship between the number of degenerated discs and imaging parameters of coronal imbalance in patients with DLS [15]. COBB angle and AVT, as local balance parameters in the coronal plane of the spine, have become one of the most used measures of scoliosis, also can be used as a predictor of DLS progression [16]. Our study found that all included patients had varying degrees of degenerated discs, this suggests a close relationship between disc degeneration and lumbar coronal instability, and indirectly confirms that disc degeneration is an important cause of DLS. The percentage of patients with degenerated discs in 2 or more segments was 95%, 19 times higher than that of patients with Single-segment lumbar disc degeneration. Meanwhile, DLS combined with degenerated discs in 3 segments was the most common, accounting for 30% of patients. 40 cases presented with degenerated discs in the L4-L5 segment, which was the most affected segment, followed by the L3-L4 segment and the L5-S1 segment. These three inferior lumbar discs showed 103 cases of degenerated discs, accounting for 76.9% of the cases, whereas only 31 cases of degenerated discs were found in the L1-L2 and L2-L3 segments. This indicates that the inferior discs are more likely to degenerate compared to other discs. And it is noteworthy that only six patients had no degenerated disc in the adjacent segments of the AV, leaving 34 patients who all had degenerated disc in the adjacent segments of the AV. In addition, Spearman’s analysis showed that there was no statistically significant relationship between the number of degenerated discs and the imaging parameters of coronal imbalance in patients with DLS, suggesting that DLS was associated with degenerated discs, but there was no statistically significant relationship between imbalance in the coronal plane of the lumbar spine and the number of degenerated discs in patients with DLS.

We believe that the frequency of degenerated discs is higher in the inferior disc occurs for two reasons: firstly, the maintenance of the stability of the lumbar spine under stress loading is dependent on the intact disc, the adjacent small joints, and the contraction of the lumbar back muscles. When the lumbar spine is subjected to compressive stress, the intervertebral disc, and the adjacent small joints are each subjected to half of the load, acting as a weight-bearing and movement-controlling mechanism, respectively, and therefore the asymmetrical stress load on the intervertebral disc and the small joints in patients with DLS can lead to lumbar instability and accelerate the development of lumbar deformities in the coronal plane [17, 18]. In addition, from a mechanical point of view, the more mobile lumbar spine is located between the less mobile parts of the thoracic spine and the pelvis, and the lower sacrum is unable to produce a corresponding coordinated cushioning action due to its poor mobility, making the lumbar spine and lumbar intervertebral discs subject to greater stress from various directions during lumbar movement. And the lower lumbar vertebrae and discs are the most concentrated areas of stress in the entire lumbar spine, therefore, lumbar movement will eventually cause a large amount of stress to be concentrated in the lower lumbar segments [19, 20]. Secondly, anatomically, the muscles and ligaments of the lower lumbar spine are more dispersed and weaker, which do not provide sufficient protection for the stability of the lumbar spine and intervertebral discs. Patients with DLS tend to have long-term poor lumbar spine use habits, and because the lower lumbar spine is prone to compression and torsion, and injury during daily life and exercise, the lower lumbar segments of patients with DLS are more prone to lumbar disc degeneration [21, 22].

Finally, considering the results of the studies we have obtained, we recommend that patients with DLS should have regular imaging examinations. We cannot ignore the coronal imbalance in patients with DLS even though the number of degenerated lumbar discs in patients with DLS is small.

## Conclusion

Our results showed an association between DLS and degenerated discs, but there was no statistically significant relationship between imbalance in the coronal plane of the lumbar spine and the number of degenerated discs in patients with DLS. The distribution of degenerated disc segments in patients with DLS showed a higher likelihood of disc degeneration in 2 or more segments, and a higher frequency of disc degeneration in the inferior disc and in the adjacent segments of the AV.

## Limitation

Firstly, this study was a single-center retrospective study with a small sample size for review compared to other similar studies. Also, as the study population was outpatients, there was a lack of information on long-term follow-up of patients, which may have biased the reliability of the findings, and a multicenter study with a large sample is needed to improve the credibility of the study.

## Data Availability

All data included in this study are available upon request by contact with the corresponding author on reasonable request.

## Abbreviations

DLS: Degenerative lumbar scoliosis
EV: End Vertebrae
CSVL: Center Sacral Vertical Line
AV: Apical Vertebrae
AVT: Apical vertebral translation
ASD: adult spinal deformity
